# A Convenient Ultraviolet Irradiation Technique for Synthesis of Antibacterial Ag-Pal Nanocomposite

**DOI:** 10.1186/s11671-016-1643-y

**Published:** 2016-09-27

**Authors:** Shuai Han, He Zhang, Lianwei Kang, Xiaoliang Li, Chong Zhang, Yongjie Dong, Shenjun Qin

**Affiliations:** 1 College of Science, Hebei University of Engineering, Handan, 056038 Hebei People’s Republic of China; 2 College of Life Science, Lanzhou University, Lanzhou, 730000 People’s Republic of China; 3 Hebei Collaborative Innovation Center of Coal Exploitation, Hebei University of Engineering, Handan, 056038 People’s Republic of China

**Keywords:** Palygorskite, Carbon dots, Ag NPs, Nanocomposite, Antibacterial activities

## Abstract

In the present work, palygorskite (Pal) was initially subjected to an ion-exchange reaction with silver ions (Pal-Ag^+^). Subsequently, Ag-Pal nanocomposites were assembled by a convenient ultraviolet irradiation technique, using carbon dots (CDs) derived from wool fiber as the reducing agent. The obtained nanocomposites were characterized by powder X-ray diffraction (XRD), ultraviolet-visible (UV-vis) spectroscopy, transmission electron microscopy (TEM), and Fourier transform infrared spectroscopy. The XRD patterns and UV-vis absorption spectra confirmed the formation of the Ag nanoparticles (NPs). Meanwhile, the TEM images showed that the Ag NPs, which exhibited sizes in the range of 3–7 nm, were located on the surface of the Pal nanofiber structures. Furthermore, the antibacterial activity of the nanocomposites was evaluated against Gram-positive (*Staphylococcus aureus*) and Gram-negative (*Escherichia coli*) bacteria by applying the disc diffusion method and minimum inhibitory concentration test. Owing to their good antibacterial properties, the Ag-Pal nanocomposites are considered to be a promising bactericide with great potential applications.

**Electronic supplementary material:**

The online version of this article (doi:10.1186/s11671-016-1643-y) contains supplementary material, which is available to authorized users.

## Background

Among the various materials used for antimicrobial activities, nanostructured materials are considered to be more effective because of their high surface-area-to-volume ratio [1–3]. Additionally, the use of inorganic nanoparticles (NPs) as antimicrobial agents has several benefits, such as improved stability and safety, compared with the use of organic antimicrobial agents [4, 5]. Typical inorganic NPs consist of Ag, Cu, ZnO, TiO_2_, SnO_2_, and CuO [6].

Ag NPs have been reported to be an effective antimicrobial agent; they have been widely used in a broad spectrum of applications including water purification, wound care, and food production [7, 8]. However, free Ag NPs without a supporting matrix might be inadequate for applications where prolonged Ag release is needed (e.g., in medical devices) [9]. On the other hand, the small size of the NPs favors their aggregation, which might cause disadvantageous changes in their chemical, physical, and antibacterial properties [10]. To overcome these issues, hybrid processes combining Ag NPs with other environmentally friendly, inert, and cheap materials are adopted to fully exploit their properties in various applications [11].

Among the possible matrices, clay minerals are particularly attractive because of their cost effectiveness and widespread availability [12, 13]. Palygorskite (Pal), a member of the clay group, is a hydrated magnesium aluminum silicate with reactive hydroxy groups on the surface [14]. Pal consists of a three-dimensional network of densely packed rods; the single rod crystal is the smallest structure unit with a length in the range of 200–500 nm and a diameter of 10–25 nm [15]. Owing to the special nanostructure, inherent stability, and large specific surface area, considerable effort has been devoted to discover new approaches of using Pal in its native and modified forms [16–18].

Composites of metal NPs supported on clay can be fabricated by using various techniques, such as ion-exchange processes, solvothermal synthesis, chemical reduction methods, and sol-gel processes [19]. However, the development of a simple and green synthetic method with environmentally friendly reagents still represents a great challenge [20]. As an emerging class of carbon nanomaterials, carbon dots (CDs) have drawn much attention due to their outstanding photostability, low environmental risk, high biocompatibility, and interesting electron transfer behavior [21–23]. In recent years, significant research efforts have been dedicated to produce noble metal NPs using CDs as the reducing agent [24, 25], providing new opportunities for the synthesis and applications of metal nanomaterials [26].

In the present work, wool fiber, a cheap and abundant bioresource, was used to fabricate wool fiber-derived CDs (WCDs) by a hydrothermal method. Then, using the obtained WCDs as a reducing agent, Ag-Pal nanocomposites were fabricated by ultraviolet (UV) radiation method, which is a simple and green process (90 min exposure to a UV lamp). The composites were then characterized by transmission electron microscopy (TEM), X-ray diffraction (XRD), and Fourier transform infrared (FTIR) spectroscopy. Finally, the antibacterial activity of the nanocomposites was evaluated using the minimum inhibitory concentration (MIC) test and Kirby-Bauer disk diffusion method. Owing to the excellent antibacterial properties against both Gram-positive (*Staphylococcus aureus*) and Gram-negative (*Escherichia coli*) bacteria, the Ag-Pal nanocomposite is considered a promising bactericide with great potential applications.

## Methods

### Materials

Pal (Jiangsu Autobang Co., Ltd., China) was treated with HCl and H_2_O_2_ to remove the impurities and activate the hydroxyl groups on the surface [27]. AgNO_3_ was obtained from Aladin Ltd. (Shanghai, China). *E. coli* (ATCC 25,922) and *S. aureus* (ATCC 25,923) were purchased from China General Microbiological Culture Collection Center (Beijing, China). Deionized (DI) water was used for the experiments.

### Methods

TEM was performed on a JEOL-2010 TEM (Japan) at 200 kV. FTIR spectra were collected within the 4000–400 cm^−1^ wavenumber range using a Nicolet 360 FTIR spectrometer with the KBr pellet technique. Nitrogen (N_2_) adsorption-desorption isotherms were measured by an ASAP 2010 analyzer with N_2_. Powder XRD (PXRD) patterns were obtained with a Rigaku-Dmax 2400 diffractometer using Cu-Kα radiation in the 2*θ* range of 5°–70°. UV-visible (UV-vis) spectra were recorded using a Perkin-Elmer Lambda 20 UV-vis spectrometer. Excitation and emission spectra were collected using a Hitachi F-4500 Fluorescence spectrophotometer. Quantitative determinations of Ag were obtained by inductively coupled plasma optical emission spectrometry (ICP-OES; IRIS Advantage ER/S spectrophotometer). The UV-vis absorption spectra were recorded on a Perkin-Elmer Lambda 950 spectrophotometer.

### Synthesis of the WCDs

WCDs were prepared by the hydrothermal treatment of wool fibers. In a typical synthesis, 0.4 g of wool fibers was transferred into a 100 mL Teflon-lined stainless steel autoclave and heated at 180 °C for a period of 12 h. When the reaction was completed, the autoclave was cooled down naturally. The resulting light yellow solution was centrifuged at 16,000 rpm for 30 min to remove the large dots, yielding a light brown aqueous solution of WCDs (yield ~36.5 %).

### Preparation of the Ag Ion-Exchanged Pal (Pal-Ag^+^)

A 25 mg of AgNO_3_ was dissolved in 25 mL of DI water. Next, 200 mg of Pal was subjected to the ion-exchange process in a AgNO_3_ solution at 25 °C for 5 h with continuous stirring. At the end of the reaction, Pal-Ag^+^ was collected by centrifugation at 8000 rpm for 30 min. The product was then dried at 80 °C overnight. It could be shown from the EDX that the concentration of elemental silver varied from 3.0 to 8.7 % for the product (Additional file 1: Figure S1), which indicated the formation of Pal-Ag^+^.

### Fabrication of the Ag-Pal Nanocomposite

Part of Pal-Ag^+^ (40 mg) was dispersed in ethanol by ultrasonic process; 4 mg of WCDs was added. Then, the mixture was placed in a UV quartz cuvette (1 × 1 × 5 cm) located 3 cm away from a 6 W UV lamp (Vilber Lourmat). After the photoreaction (254 nm, 50 min), the color of the solution changed from light yellow to dark brown, implying the formation of the Ag-Pal nanocomposite (Additional file 2: Figure S2).

### Antimicrobial Tests

*E. coli* and *S. aureus* were cultivated in a beef extract peptone medium at 37 °C for 12 h with a shaking incubator. The disk diffusion test was performed according to a reported procedure [28], while equal amounts of Pal-Ag^+^ and Ag-Pal nanocomposites (200 ± 10 μg) were loaded into the filter papers. The bacterial suspension (100 μL of 10^4^–10^5^ CFU mL^−1^) was applied uniformly on the surface of the nutrient agar plates. Then, the disks were placed on the plates and incubated at 35 °C for 24 h. After the incubation period, the zones of inhibition were measured and digital images of the plates were captured. For the minimum inhibitory concentration (MIC) test, the survival of the organisms was observed by visual inspection as recorded [29].

### Leaching Test

To evaluate the stability of the Ag-Pal nanocomposite, leaching tests were performed. Typically, 0.2 g of nanocomposite was dispersed in 20 mL of DI water and vigorously shaken in a shaking thermostatic bath (30 °C, 200 rpm) for various time periods. After shaking, the suspensions were centrifuged at 8000 rpm for 20 min. The supernatant was analyzed using ICP-OES to establish the quantity of Ag leached into the water.

## Results and Discussion

### Characterization of WCDs

**Fig. 1:**
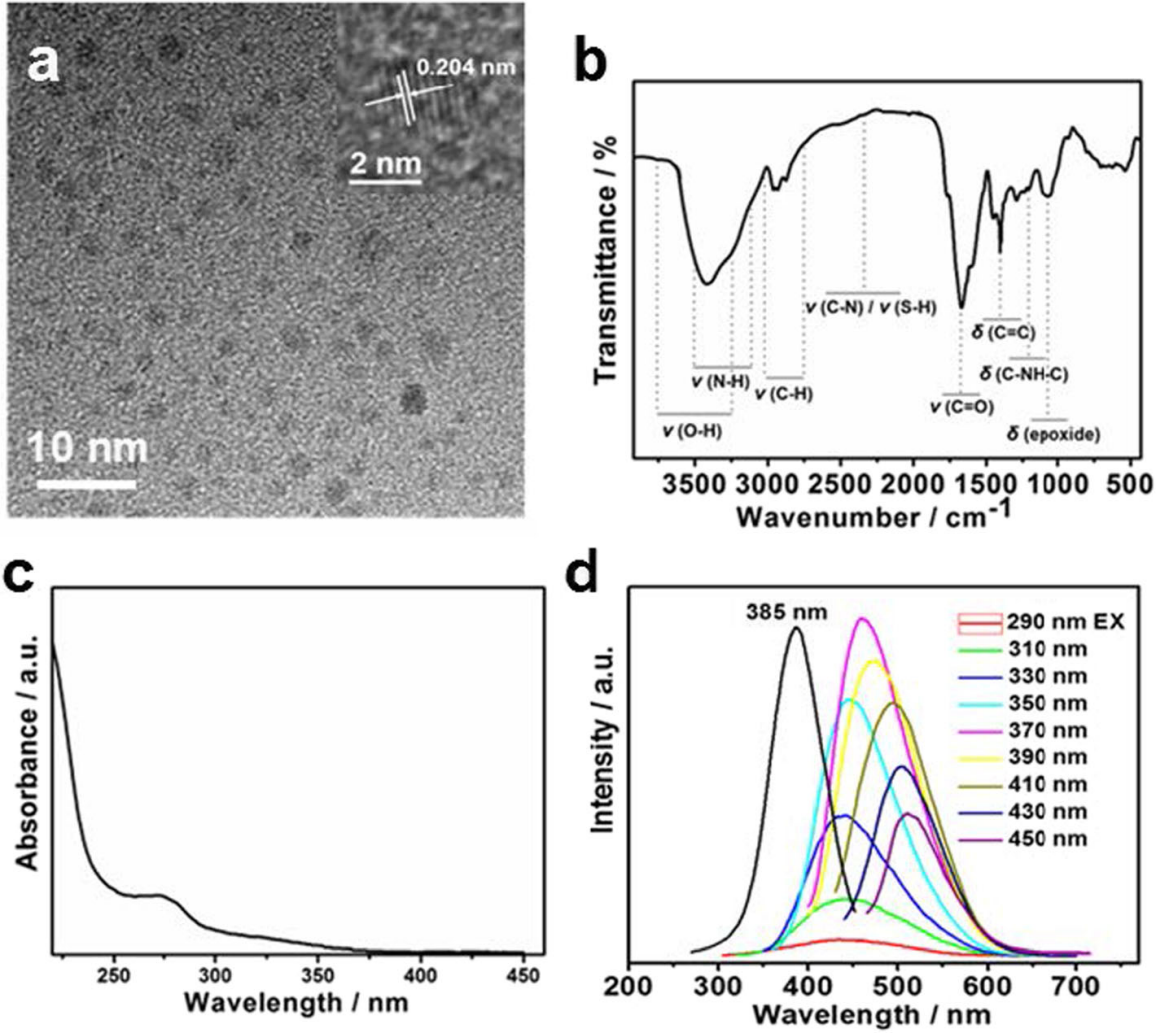
**a** Transmission electron microscopy (TEM) image (*inset*: high-resolution TEM image), **b** Fourier transform infrared spectrum, **c** ultraviolet-visible absorption spectra, and **d** excitation-dependent photoluminescence spectra of WCDs; the excitation was monitored at the maximum emission peak of 455 nm (*black line*)

The low-resolution TEM image (Fig. 1a) shows that the as-synthesized WCDs are uniform in size (average diameter of 1–5 nm) and exhibit a nearly spherical shape. The high-resolution TEM image reveals the lattice fringes of WCDs to be 0.206 nm (Fig. 1a), which corresponds to the [102] facet of graphitic (sp^2^) carbon [30]. The FTIR spectra of the WCDs are shown in Fig. 1b. The peak between 3200 and 3700 cm^−1^ is assigned to the stretching of the O–H and N–H bonds, while the peaks at 2923 and 2850 cm^−1^ are ascribed to the stretching of the C–H bond. The peak at 2350 cm^−1^ is assigned to the stretching of O–H and C–N bonds. The peaks at 1635, 1570, and 1420 cm^−1^ can be identified as the stretching of the C=O group, the bending of the N–H bond, and the stretching of the C=C group, respectively [31–33]. Owing to the aromatic π system and the n–p* transition of the carbonyl, the UV-vis spectrum of the obtained WCDs (Fig. 1c) exhibits a broad absorption band centered at 274 nm [34]. Similar to the previous reports, excitation-dependent photoluminescence (PL) was also observed for the WCDs (Fig. 1d) [35].

### Characterization of the Ag-Pal Nanocomposite

**Fig. 2:**
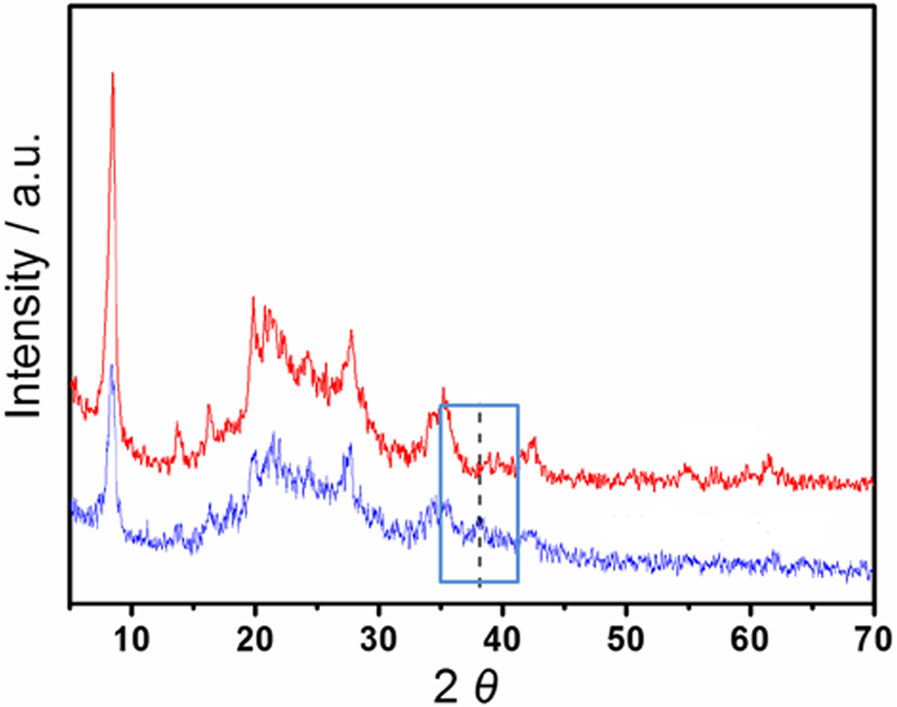
Comparison of powder X-ray diffraction patterns of Pal and Pal nanocomposite

The PXRD patterns of Pal and the Ag-Pal nanocomposite are shown in Fig. 2. The Pal characteristic peaks appear at 2*θ* values of 8.5, 19.7, 26.6, 34.5, and 42.3°, which are consistent with those in other reports [36]. For the Ag-Pal nanocomposite, the peaks are evident and almost identical (Fig. 2). This phenomenon indicates that the structure of Pal was maintained after the modification. However, a new peak was observed corresponding to the formation of Ag NPs in the nanocomposite. The peak appeared at 2*θ* = 38° and can be attributed to the (111) crystallographic planes of the face-centered cubic Ag crystals [37]. The UV-vis absorption spectrum of the Ag-Pal nanocomposite is shown in Additional file 3: Figure S3. Notably, an absorption band with a maximum at 415 nm, corresponding to the spectral behavior of Ag crystals, can be observed, supplying new evidence of the formation of Ag NPs [38].

**Fig. 3:**
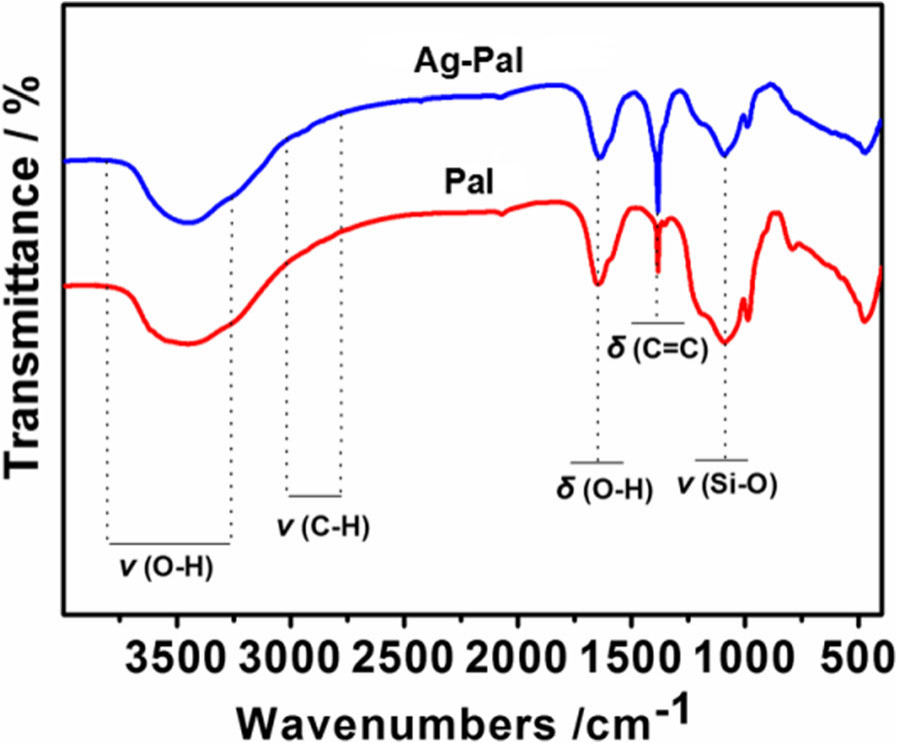
Fourier transform infrared contrastive analysis of Pal and Ag-Pal nanocomposite

The FTIR spectra of Pal and Ag-Pal nanocomposite are shown in Fig. 3. The most intense band in the FTIR spectra of Pal appeared at 1043 cm^−1^ and was attributed to the Si–O in-plane stretching vibration. The broad bands at 1645 and 3456 cm^−1^ were attributed to the bending and stretching vibrations, respectively, of the hydroxyl groups of the water molecules present in the clay [39]. However, all the bands in the Ag-Pal nanocomposite spectrum decreased in intensity, compared to those of the Pal matrix, indicating that the Ag NPs interacted with the surface of the Pal support. On the other hand, a clear increase of the intensity of the band at 1420 cm^−1^ was observed, which might be attributed to the stretching of the WCD C=C bond in the nanocomposites.

**Fig. 4:**
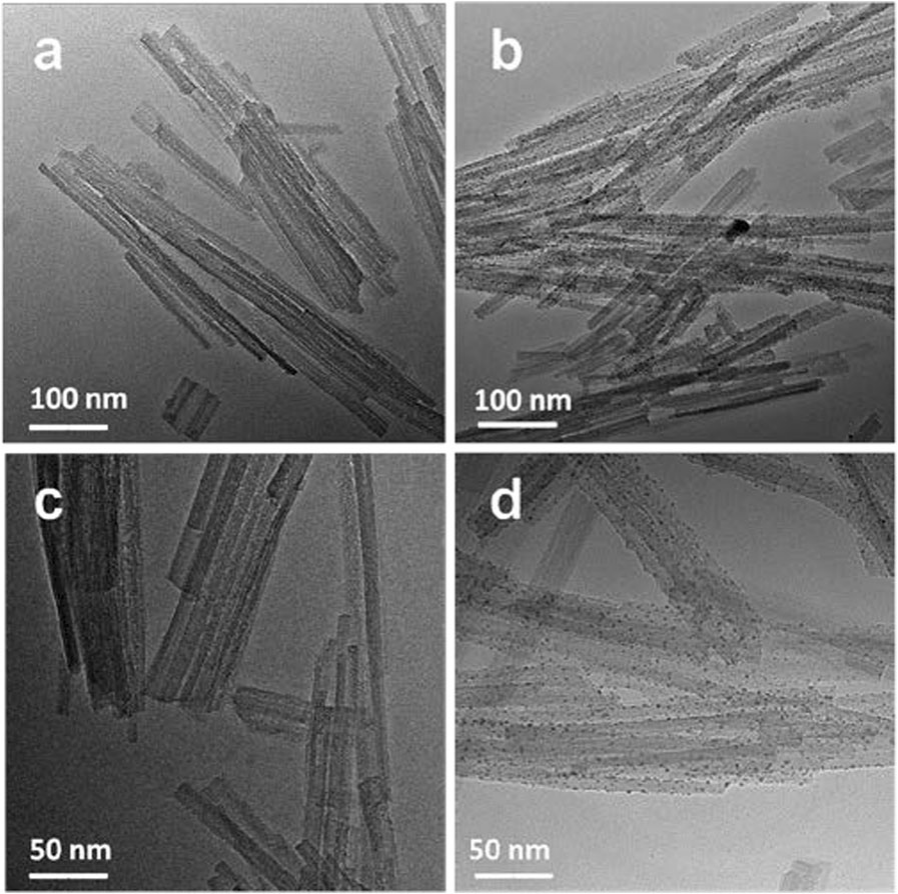
TEM images of the Pal (**a**, **c**) and the Ag-Pal nanocomposite (**b**, **d**)

**Fig. 5:**
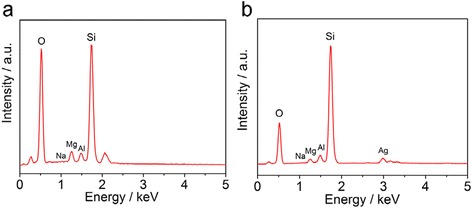
EDX spectrum of Pal (**a**) and Ag-Pal nanocomposite (**b**)

The TEM micrographs of Pal and Ag-Pal are shown in Fig. 4. After the modification, many small spherical NPs appeared on the surface of the Pal nanofiber structures (Fig. 4b, d); according to the XRD and FTIR results, these particles were identified as Ag NPs. The mean diameter of the Ag NPs was determined to be ~3–7 nm. Energy dispersive X-ray spectroscopy (EDX) was further used to analyze the elemental constitution of the Pal and Ag-Pal (Fig. 5). It is shown from Fig. 5a that silicon, magnesium, and aluminum were the principal elements present for the Pal. After the ultraviolet radiation reaction, new peak at 3.9 keV was observed corresponding to the silver element in the nanocomposite (Fig. 5b). It could be also obtained from the EDX that the concentration of elemental silver varied from 3.2 to 8.8 % for the Ag-Pal nanocomposite.

### Antibacterial Properties of the Ag-Pal Nanocomposite

**Fig. 6:**
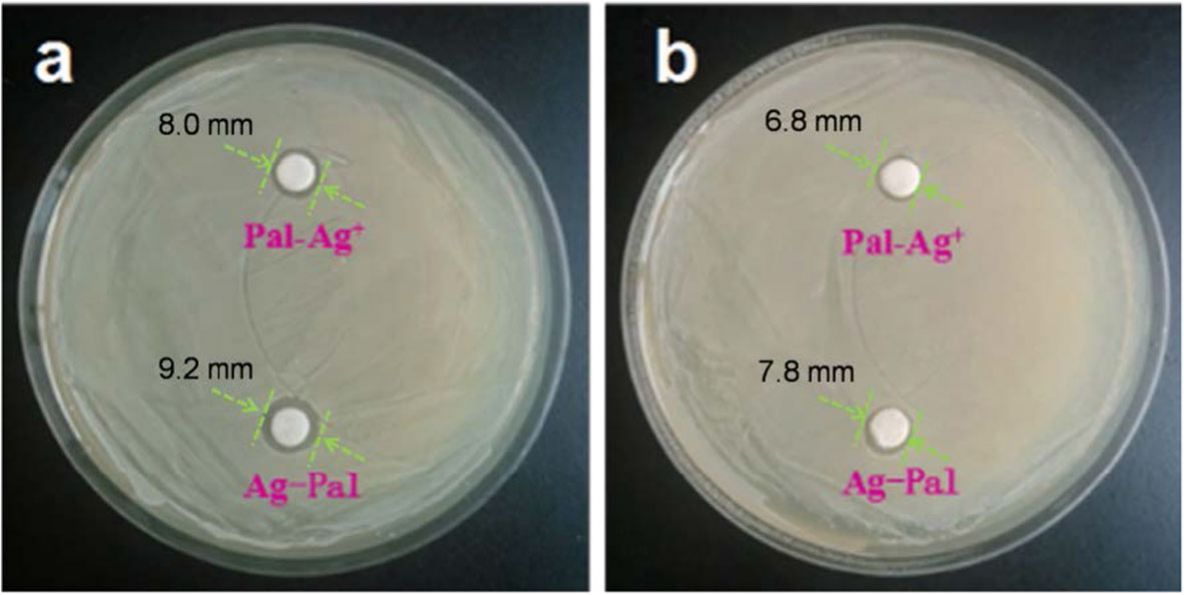
Representative images of agar plates containing Pal-Ag^+^ and Ag-Pal impregnated disks and diameter of inhibition zone for **a** *S. aureus* and **b** *E. coli*

The antibacterial properties of the Ag-Pal nanocomposite against Gram-negative (*E. coli*) and Gram-positive (*S. aureus*) bacteria were examined using the disk diffusion method and MIC test; Pal-Ag^+^ was used as the contrast material for the comparison of the activity. As shown in Fig. 6a, b, the disks with Ag-Pal were surrounded by a larger inhibition zone than the disks with Pal-Ag^+^ for both *E. coli* and *S. aureus* strains. Meanwhile, the MIC of Ag-Pal was lower than that of Pal-Ag^+^ after 24-h incubation (Additional file 4: Table S1). Thus, compared with Pal-Ag^+^ at the same Ag concentration, Ag-Pal exhibited a superior antimicrobial activity. Although Pal-Ag^+^ could slowly release Ag^+^ ions as the antimicrobial agent, the diffusion of Ag^+^ ions might have been hindered by the formation of secondary compounds, such as AgCl in the cultivation medium. In the case of Ag-Pal, the slowly released Ag NPs could freely diffuse into the cultivation medium and act as biocidal agents [24]. Therefore, the synthesized Ag-Pal nanocomposite showed a greater antibacterial activity than Pal-Ag^+^.

### Leaching Tests

As one of the most important applications of the studied nanocomposite is the treatment of drinking water, the stability of Ag-Pal in water was evaluated. In the leaching test, vigorous agitation is adopted to ensure that the material is stable under any circumstances occurring in the industrial process. Table 1 shows that the concentration of Ag in the supernatant was <0.015 mg/L after 12 h, which could be attributed to the stable attachment of the Ag NPs to the Pal matrix. Meanwhile, the obtained results indicate that the nanocomposite is harmless in the case of drinking water treatment, as the amount of leached Ag was significantly lower than the maximum allowable concentration (0.1 mg/L, approved by the World Health Organization) [40]. So, in the water treating process, the Pal-Ag nanocomposite could be used as the excellent contact fungicide when dispersed in the water. Then, clean drinking water could be obtained when the nanocomposite was separated out.

**Table 1:** Leaching test results of the Ag-Pal nanocomposite

Sample		0 min	30 min	60 min	4 h	12 h
Ag-Pal	Ag	<0.005	<0.005	<0.005	<0.005	0.015

## Conclusions

In summary, Ag-Pal nanocomposite was assembled by a rapid and facile UV radiation method (*λ* = 254 nm), using CDs derived from wool fiber as a reducing agent. The PXRD spectra and TEM observations confirmed the formation of Ag NPs on the clay surfaces. The antibacterial activity tests revealed that the nanocomposite has a good biocidal effect on both Gram-positive (*S. aureus*) and Gram-negative (*E. coli*) strains, while the leaching tests showed that the nanocomposite remained stable under vigorous agitation. Thus, the obtained Ag-Pal nanocomposite is considered to be a promising bactericide with great potential applications.

## Additional Files

Additional file 1: Figure S1. EDX spectrum of Pal-Ag^+^. (DOCX 457 kb) Additional file 2: Figure S2. Flow chart of Ag-Pal synthesis as a function of UV irradiation time. (DOCX 229 kb) Additional file 3: Figure S3. UV-vis absorption spectra of Ag-Pal nanocomposite in ethanol solution. (DOCX 46 kb) Additional file 4: Table S1. Minimum inhibitory concentration of Pal-Ag^+^ and Ag-Pal nanocomposite for two microorganisms. (DOCX 16 kb)
