# Supplementary figures and images for: A Convenient Ultraviolet Irradiation Technique for Synthesis of Antibacterial Ag-Pal Nanocomposite
Source: Nanoscale Res Lett. 2016 Sep 27;11:431. doi: 10.1186/s11671-016-1643-y (PMC5039142; doi:10.1186/s11671-016-1643-y)

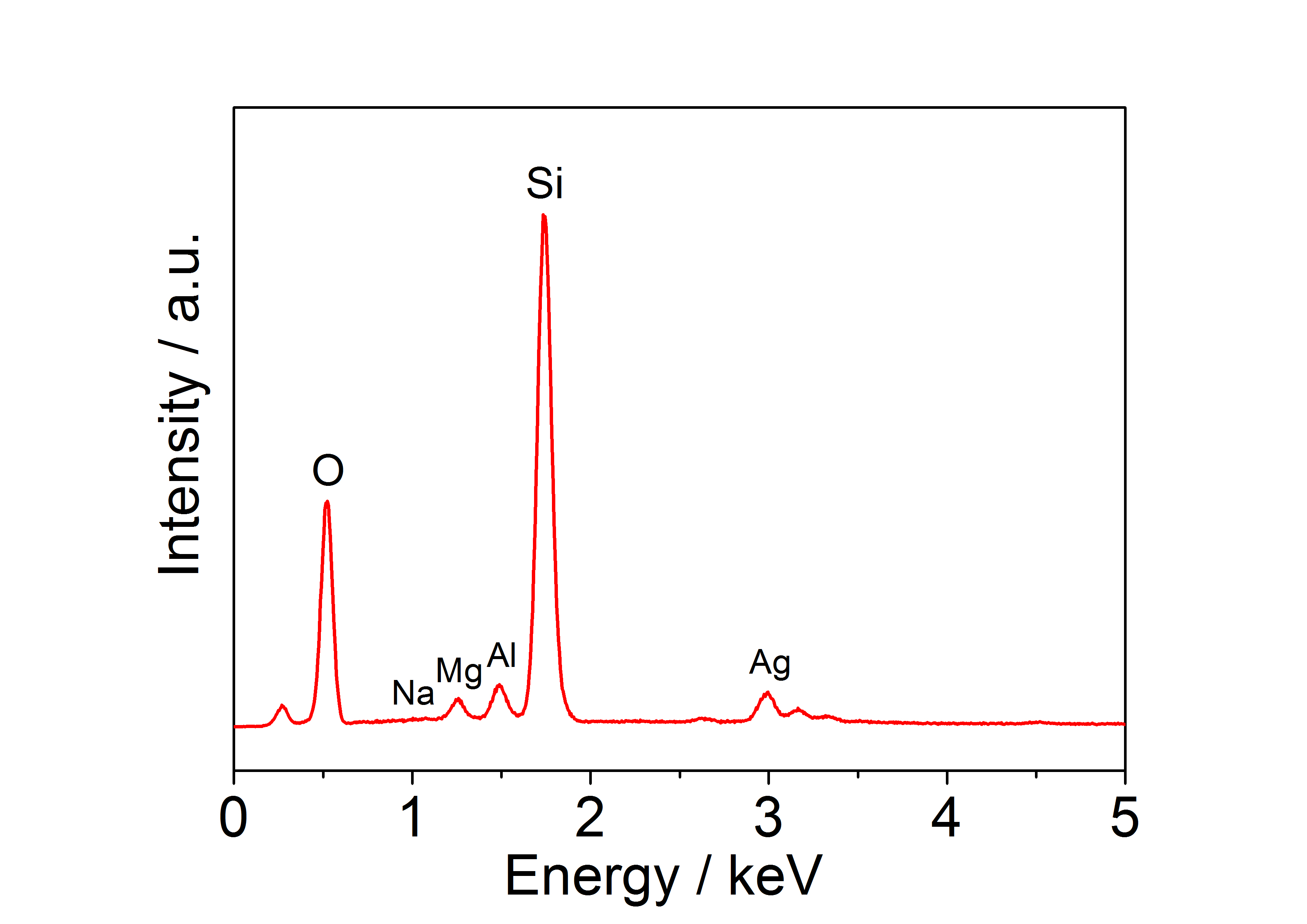


Fig. S1. EDX spectrum of Pal-Ag+

Supplement: Additional file 1: Figure S1. — EDX spectrum of Pal-Ag+. (DOCX 457 kb) [file 11671_2016_1643_MOESM1_ESM.docx]

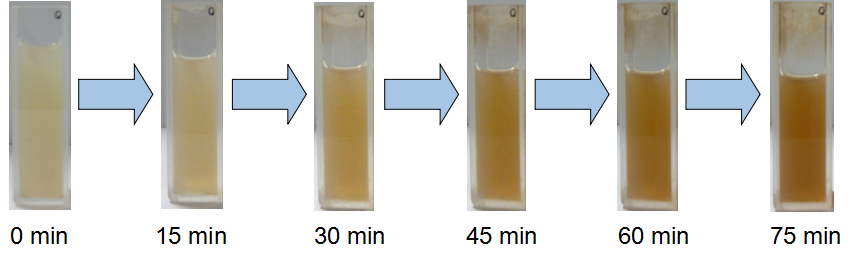


Fig. S2 Flow chart of Ag-Pal synthesis as a function of UV irradiation time.

Supplement: Additional file 2: Figure S2. — Flow chart of Ag-Pal synthesis as a function of UV irradiation time. (DOCX 229 kb) [file 11671_2016_1643_MOESM2_ESM.docx]
