# Supplementary material for: A Convenient Ultraviolet Irradiation Technique for Synthesis of Antibacterial Ag-Pal Nanocomposite
Source: Nanoscale Res Lett. 2016 Sep 27;11:431. doi: 10.1186/s11671-016-1643-y (PMC5039142; doi:10.1186/s11671-016-1643-y)
Supplement: Additional file 3: Figure S3. — UV-vis absorption spectra of Ag-Pal nanocomposite in ethanol solution. (DOCX 46 kb) [file 11671_2016_1643_MOESM3_ESM.docx]

Fig. S3. UV-vis absorption spectra of Ag-Pal nanocomposite in ethanol solution.
