# Supplementary material for: A Convenient Ultraviolet Irradiation Technique for Synthesis of Antibacterial Ag-Pal Nanocomposite
Source: Nanoscale Res Lett. 2016 Sep 27;11:431. doi: 10.1186/s11671-016-1643-y (PMC5039142; doi:10.1186/s11671-016-1643-y)
Supplement: Additional file 4: Table S1. — Minimum inhibitory concentration of Pal-Ag+ and Ag-Pal nanocomposite for two microorganisms. (DOCX 16 kb) [file 11671_2016_1643_MOESM4_ESM.docx]

**Table S1** Minimum inhibitory concentration of Pal-Ag^+^ and Ag-Pal nanocomposite for two microorganisms.

| Culture | Strain no. | MIC_a_ | MIC_b_ |
| --- | --- | --- | --- |
| S. aureus | ATCC 25,922 | 80 | 50 |
| E. coli | ATCC 25,923 | 140 | 110 |

*Notes: MIC_a_, minimum inhibitory concentration (μg mL^−1^) of Pal-Ag^+^, MIC_b_, minimum inhibitory concentration (μg mL^−1^) of Ag-Pal nanocomposite.*
